# Mounier-Kuhn syndrome: an unusual cause of bronchiectasis

**DOI:** 10.1590/0100-3984.2017.0167

**Published:** 2019

**Authors:** Rômulo Florêncio Tristão Santos, Tiago Kojun Tibana, Isa Félix Adôrno, Edson Marchiori, Thiago Franchi Nunes

**Affiliations:** 1 Universidade Federal de Mato Grosso do Sul (UFMS), Campo Grande, MS, Brazil.; 2 Universidade Federal do Rio de Janeiro (UFRJ), Rio de Janeiro, RJ, Brazil.

**Figure 1:**
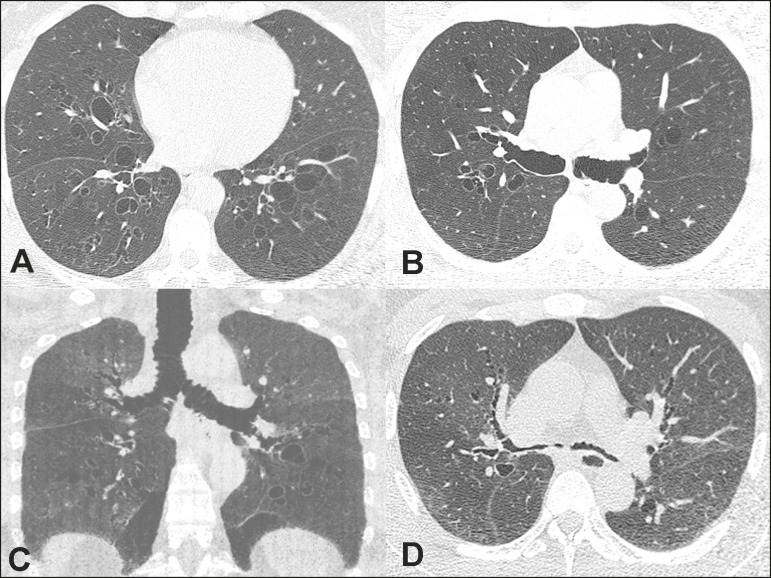
**A,B:** CT scan of the chest, acquired during inspiration, showing bilateral bronchiectasis, together with marked dilatation of the main bronchi. **C:** Coronal reconstruction showing, in addition to the bronchiectasis, dilatation of the main bronchi and the trachea. **D:** Slice acquired during expiration, showing near-total collapse of the bronchial tree.

Dear Editor,

A 49-year-old nonsmoking man presented with a six-year history of recurrent infections of the lower respiratory tract, having been asymptomatic between the episodes. The initial physical examination showed that the patient was in good general health, with a blood pressure of 120/80 mmHg, normal cardiac auscultation findings, a heart rate of 77 bpm, pulmonary auscultation showing sparse rales, a respiratory rate of 18 breaths/min, oxygen saturation of 93% on room air, and a normal abdomen. A computed tomography (CT) scan of the chest, acquired during inspiration (Figures 1A, 1B, and 1C), showed dilatation of the trachea and main bronchi (transverse diameter of 3.5 cm and 1.8 cm, respectively), as well as bronchiectasis in the middle and lower regions of both lungs. A slice acquired during expiration (Figure 1D) demonstrated partial collapse of the trachea and main bronchi. The pattern seen on CT was considered to be diagnostic of tracheobronchomegaly. Spirometry produced the following results before and after bronchodilator administration, respectively: FVC, 87% and 88%; FEV_1_, 69% and 75%; FEV_1_/FVC ratio, 64% and 69%; and FEF, 44% and 52%. The patient was treated with corticosteroids and bronchodilators. At this writing, he is in outpatient follow-up.

The importance of tracheobronchial diseases has been emphasized in recent studies^(^^1^^-^^4^^)^. Tracheobronchomegaly, or Mounier-Kuhn syndrome, is a rare disease, observed mainly in middle-aged men before the fifth decade of life^(^^5^^)^, that is characterized by atrophy or the absence of elastic fibers or smooth muscle in the wall of the trachea and the main bronchi, resulting in dilatation of those structures^(^^6^^-^^8^^)^. It is believed that the weakness of connective tissue, associated with the inhalation of air pollutants and smoking, is the main factor in the development of this condition^(^^6^^)^. Because of those anatomical and physiological changes, the flaccid airways widen during inspiration and collapse during expiration^(^^6^^)^; in addition to that dynamic change, bronchial or tracheal diverticulosis and bronchiectasis are common^(^^7^^,^^8^^)^.

The clinical presentation of Mounier-Kuhn syndrome is nonspecific, including the accumulation of secretions, with productive cough, dyspnea, and recurrent infections of the lower respiratory tract. In rare cases, there can be hemoptysis or pneumothorax^(^^6^^-^^8^^)^. The diagnosis is made on the basis of imaging findings. The most sensitive examination is CT of the trachea and main bronchi, with images acquired during inspiration and expiration^(^^7^^,^^8^^)^. The treatment is generally supportive, including respiratory therapy, appropriate antibiotic therapy for the recurrent infections, and smoking cessation^(^^9^^,^^10^^)^.

In conclusion, Mounier-Kuhn syndrome should be considered in patients who present with bronchiectasis accompanied by abnormal dilatation of the trachea and the main bronchi. The preferred diagnostic imaging method is CT of the chest.
